# Alzheimer’s Disease: From Genetic Variants to the Distinct Pathological Mechanisms

**DOI:** 10.3389/fnmol.2017.00319

**Published:** 2017-10-06

**Authors:** Qiying Sun, Nina Xie, Beisha Tang, Rena Li, Yong Shen

**Affiliations:** ^1^Department of Geriatric Neurology, Xiangya Hospital, Central South University, Changsha, China; ^2^Center for Advanced Therapeutic Strategies for Brain Disorders and Center for Hormone Advanced Science and Education, Roskamp Institute, Sarasota, FL, United States; ^3^National Clinical Research Center for Mental Disorders, Beijing Key Laboratory of Mental Disorders, Beijing Anding Hospital, Capital Medical University, Beijing, China; ^4^Beijing Institute for Brain Disorders, Capital Medical University, Beijing, China; ^5^Neurodegenerative Disorder Research Center, University of Science and Technology of China School of Life Sciences, Hefei, China; ^6^Hefei Material Science at Microscale National Laboratory, Hefei, China

**Keywords:** Alzheimer’s disease, genetics, mechanism, GWASs, EOAD, LOAD

## Abstract

Being the most common cause of dementia, AD is a polygenic and neurodegenerative disease. Complex and multiple factors have been shown to be involved in its pathogenesis, of which the genetics play an indispensable role. It is widely accepted that discovery of potential genes related to the pathogenesis of AD would be of great help for the understanding of neurodegeneration and thus further promote molecular diagnosis in clinic settings. Generally, AD could be clarified into two types according to the onset age, the early-onset AD (EOAD) and the late-onset AD (LOAD). Progresses made by genetic studies on both EOAD and LOAD are believed to be essential not only for the revolution of conventional ideas but also for the revelation of new pathological mechanisms underlying AD pathogenesis. Currently, albeit the genetics of LOAD is much less well-understood compared to EOAD due to its complicated and multifactorial essence, Genome-wide association studies (GWASs) and next generation sequencing (NGS) approaches have identified dozens of novel genes that may provide insight mechanism of LOAD. In this review, we analyze functions of the genes and summarize the distinct pathological mechanisms of how these genes would be involved in the pathogenesis of AD.

## Introduction

Being the most common cause of dementia, AD is a polygenic and neurodegenerative disease, defined as the presence of extracellular amyloid plaques and intracellular neurofibrillary tangles (Ramirez-Bermudez, 2012). Neuroinflammation, synaptic and neurotransmitter loss are also involved in the pathogenesis of AD (Huang and Mucke, 2012; Anand et al., 2014). Clinically, patients’ increasingly loss of memory and impairment of related cognitive functions is the main feature of AD, which can be further divided into two subtypes, the early onset and late-onset forms, based on the on-set age. (Reitz et al., 2011).

Early-onset AD (EOAD) is usually autosomal dominant inherited, constituting barely 1–2% of AD, with genes including *amyloid precursor protein (APP), presenilin 1 (PSEN1)*, and *presenilin 2 (PSEN2)* being regarded as major factors (Reitz et al., 2011; Alzheimer’s Association, 2015). Although, late-onset AD (LOAD) is epidemiologically more common compared to EOAD, it is much more complex genetically because of the involvement of genetic, epigenetic and environmental factors. The *apolipoprotein E* (*APOE*) 𝜀4 allele is the first discovered genetic risk factor for LOAD (Liu et al., 2013). Thereafter, with the advent of the genome-wide association studies (GWASs), dozens of additional genes have been found as potential risk factors for LOAD. This long gene list has already included *ABCA7, BIN1, CASS4, CD2AP, CD33, CELF1, CLU, CR1, DSG2, EPHA1, FERMT2, HLA-DRB5/DRB1, INPP5D, MEF2C, MS4A4*/*MS4A6E, NME8, PICALM, PTK2B, SLC24A4*/*RIN3, SORL1, ZCWPW1* (Harold et al., 2009; Lambert et al., 2009; Seshadri et al., 2010; Hollingworth et al., 2011; Naj et al., 2011; Lambert et al., 2013; Dong et al., 2017), with novel identified genes, such as *TREM2* and *PLD3* which might be involved in LOAD, continuously being added (Guerreiro et al., 2013; Jonsson et al., 2013; Cruchaga et al., 2014). The discovery of these genes has facilitated our gaining of the in-depth knowledge of the signaling pathways participated in AD pathogenesis. In this review, we will analyze functions of these genes and summarize possible mechanisms of how these genes would be involved in the pathogenesis of AD.

### Early-Onset Alzheimer’s Disease (EOAD)

#### Amyloid β (Aβ) Metabolism

Highly penetrant mutations in *APP, PSEN1, PSEN2*, cause the autosomal dominant EOAD (Reitz et al., 2011; Alzheimer’s Association, 2015). Additionally, rare variants in *APP, PSEN1, PSEN2* (Cruchaga et al., 2012), and *ADAM10* (Kim et al., 2009), have been listed as the risk factors for LOAD (Panza et al., 2012). These studies indicated that the disturbance of Aβ metabolism plays a central role in AD pathogenesis.

#### APP

The *APP* gene is located on chromosome 21 and contains 19 exons for encoding a ubiquitously expressed type I transmembrane protein amyloid precursor protein (APP) (Goldgaber et al., 1987). The amyloidogenic pathway and non-amyloidogenic pathway are the two mutually exclusively pathways thought to be involved. The amyloidogenic pathway is defined as consecutive cleavage of APP by β- and γ-secretase. Aβ, soluble APP ectodomain (sAPPβ) and the APP intracellular domain (AICD) are the generated products (O’Brien and Wong, 2011; Zhang et al., 2011). Alternatively, α- and γ-secretase are engaged in the non-amyloidogenic pathway. Soluble APP ectodomain (sAPPα), p3-peptide and AICD are the end-products (O’Brien and Wong, 2011; Zhang et al., 2011).

Goate et al. (1991) first discovered a missense mutation in *APP* in AD pedigrees. At least 40 *APP* mutations are known to cause familial AD, mainly with an autosomal dominant inheritance pattern^1^. Two recessive mutations in *APP*, E693Δ and A673V, were also identified to cause EOAD (Di Fede et al., 2009; Giaccone et al., 2010). Most of these mutations are found in the neighborhood of the Aβ domain (exons 16 and 17 of *APP*). The Swedish *APP* mutation (KM670/671NL) lies at the N-terminus of the Aβ domain and increases plasma Aβ levels by 2 to 3-fold by affecting the efficiency of β-secretase cleavage (Mullan et al., 1992). A sensible hypothesis is that excessive production of Aβ surpassing a certain threshold may cause AD. A supporting phenomenon is that Down syndrome patients, who have an extra copy of *APP* due to the 21 chromosome triplet, usually develop AD in their early life (Zekanowski and Wojda, 2009). Other *APP* mutations cluster at or after the C-terminal amino acids of the Aβ domain, such as the Flemish mutation (A692G) (Hendriks et al., 1992), Italian mutation (E693K) (Zou et al., 2014), Dutch mutation (E693Q) (Levy et al., 1990), Arctic mutation (E693G) (Kamino et al., 1992), and Iowa mutation (D694N) (Grabowski et al., 2001), Iranian mutation (T714A) (Pasalar et al., 2002), Australian mutation (T714I) (Kumar-Singh et al., 2000; Bornebroek et al., 2003), French mutation (V715M) (Ancolio et al., 1999; Bornebroek et al., 2003), German mutation (V715I) (Cruts et al., 2003), Florida mutation (I716V) (Eckman et al., 1997), and London mutation (V717I) (Goate et al., 1991). One thing these mutations may have in common is that they could produce more Aβ42 while decreasing the production of Aβ40 by affecting the cleaving activity of γ-secretase. Since Aβ42 is more amyloidogenic and easier to aggregate than Aβ40, patients with such *APP* mutations are more susceptible to AD, although their total amount of Aβ seems to be at the normal level. The Arctic mutation, E693G, affects neither the total Aβ amount nor the ratio of Aβ42 to Aβ40 (Kamino et al., 1992). However, this mutation increases the aggregation rate of the mutant peptide. These findings altogether indicate Aβ aggregation plays a key role in AD pathogenesis.

#### *PSEN1* and *PSEN2*

*PSEN1* and *PSEN2* are located at chromosome 14q24.3 and 1q31-q42, respectively, encoding the presenilin 1 and presenilin 2 proteins, which are participated in the formation of γ-secretase complex (Steiner et al., 2008). In 1995, the first batch of mutations of the two genes were identified by researchers in EOAD families (Levy-Lahad et al., 1995; Rogaev et al., 1995; Sherrington et al., 1995). To date, 219 different *PSEN1* mutations and 16 *PSEN2* mutations have been identified in association with EOAD^1^. *PSEN1* mutations account for 80% of the early-onset familial AD (EOFAD) cases, with *PSEN2* mutations found in 5% EOFAD families^1^.

In the APP cleavage scenario, endoproteolysis at the C-terminal end followed by a second cleavage at the N-terminal end of the Aβ domain was executed by the γ-secretase, resulting in the generation of Aβ fragments (O’Brien and Wong, 2011; Zhang et al., 2011). Normally, most of the Aβ fragments are the less amyloidogenic Aβ40, Aβ42 occupies a small percentage. In contrast, the mutant γ-secretase would predominantly yield Aβ42 with small amount of Aβ40. Similar to what we have described for *APP* mutations, patients baring mutations of *PSEN1* or *PSEN2* might also be more susceptible to AD due to accumulation of the more amyloidogenic protein Aβ42 (Bagyinszky et al., 2014).

#### ADAM10

Recently, having worked through 1000 LOAD families, researchers found, Q170H and R181G, in 7 pedigrees of them (Kim et al., 2009). *ADAM10* gene is located at chromosome 15q21.3, and encodes the AMAD10 protein, which is a member of the disintegrin and metalloprotease family (Saftig and Lichtenthaler, 2015). ADAM10 has been shown not only be able to readjust the constitutive activity of α-secretase, but to be responsible for accommodation of the regulatable activity of α-secretase in APP cleavage (Lammich et al., 1999; Lopez-Perez et al., 2001; Saftig and Lichtenthaler, 2015). Both Q170H and R181G mutations reside in the ADAM10 prodomain and significantly damage the cleavage ability of ADAM10 at the β-secretase site of APP both *in vitro* and *in vivo* (Kim et al., 2009). These findings further support the hypothesis that alteration of APP processing and Aβ generation is sufficient to cause AD.

**FIGURE 1:**
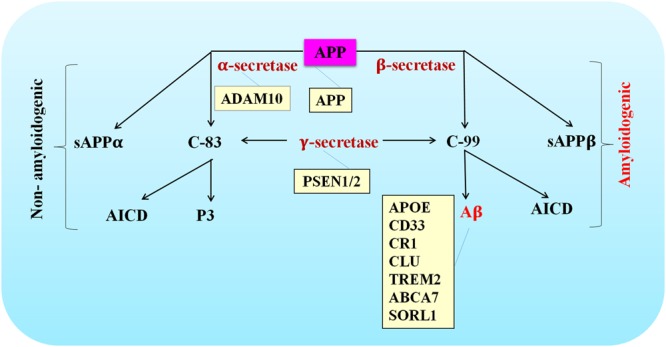
Schematic of APP processing pathways that are either amyloidogenic or non-amyloidogenic. The site of action of various AD-associated mutations are listed in the orange colored boxes.

Since Aβ peptides were discovered as a major pathological feature in AD brains, the hypothesis that excessive accumulation of misfolded β-sheet proteins causes AD started to gain public recognition. More and more evidence highlighted by genetic studies has been reported to support the central role that Aβ played in the pathogenesis of AD. For example, highly penetrant mutations have been identified as risk factors of AD in genes whose translation products are involved in APP processing and Aβ generation. Mutated genes such as *APP, PSEN1*, and *PSEN2* are thought to contribute to the pathogenesis of EOAD, while rare variants in *ADAM10* may increase the risk of developing LOAD. Given Aβ production was affected by mutations or variants in these genes, these findings further strengthened causal relationship between Aβ generation and AD pathogenesis (**Figure 1**).

### Late-Onset for Alzheimer’s Disease (LOAD)

#### Cholesterol Metabolism

The *APOE* 𝜀4 allele has been identified as a main risk factor for LOAD (Michaelson, 2014). The encoded protein apolipoprotein E (ApoE) plays the role as a cholesterol carrier in the brain. This implicates the role of cholesterol metabolism pathway in AD pathogenesis. Additionally, GWAS studies have identified several genes that might be potential risk factors for LOAD, including *ABCA7, CLU*, and *SORL1* (Harold et al., 2009; Lambert et al., 2009; Hollingworth et al., 2011; Lambert et al., 2013; Dong et al., 2017), which are involved in cholesterol metabolism.

#### APOE

The *APOE* is a gene situated in chromosome 19q13.2 encoding a protein containing 299 amino acids which is mainly expressed in the liver and brain (Siest et al., 1995). APOE is a key component of the lipoprotein complexes and plays a role in cholesterol metabolism by regulating cholesterol transport, delivery and distribution (Mahley and Rall, 2000; Lambert et al., 2009; Alonso Vilatela et al., 2012). *𝜀2, 𝜀3*, and *𝜀4* are the are three common alleles of *APOE* in humans differed in sequence by two single nucleotide polymorphisms, rs429358 and rs7412 (amino-acid position 112 and 158) in exon 4 (Liu et al., 2013; Michaelson, 2014). *APOE 𝜀3* allele is the most frequent isoform and accounts for 50–90% in all populations (Mahley and Rall, 2000; Alonso Vilatela et al., 2012). The percentage of individuals having the *APOE 𝜀4* allele is approximately 50% in LOAD patients compared with 20–25% in controls (Alonso Vilatela et al., 2012; Michaelson, 2014). *So far APOE 𝜀4* is the most well-established genetic risk factor for both sporadic LOAD and familial AD in different populations (Harwood et al., 1999; Quiroga et al., 1999; Evans et al., 2000). Compared with controls having no *𝜀4* alleles, the risk of AD is 4 times higher when subjects bearing one copy of the *𝜀4* allele, and 12 times higher with two copies (Alonso Vilatela et al., 2012). Conversely, the lower prevalence of the *𝜀2* allele in AD individuals compared with controls implicates its protective role in AD (Alonso Vilatela et al., 2012; Michaelson, 2014). In addition, the *APOE 𝜀4* allele can affect clinical diagnosis of AD by influencing MRI features except white matter lesion volume (Biffi et al., 2010).

The mechanism of *APOE* increasing AD risk is not well known. The different APOE isoforms have different effects on Aβ aggregation and clearance in AD pathogenesis (Castellano et al., 2011). Clearance of Aβ in the brain depends on coordination with APOE (Wollmer, 2010; Verghese et al., 2013). Specifically, types of APOE that Aβ bound to affect its transportation efficiency. Aβ being bound to APOE2 or APOE3 results in better efficiency compared to APOE4 (Wollmer, 2010). APOE4 can also participate in other pathways, such as neuronal glucose hypometabolism, mitochondrial abnormalities and oxidative stress, by which play an important role in AD pathogenesis (Liu et al., 2013; Huang and Mahley, 2014).

#### ABCA7

*ABCA7* is a gene situated in chromosome 19p13.3 encoding ATP-binding cassette transporter A7 (ABCA7) which is a member of the ABC superfamily (Kim et al., 2006). The protein is highly expressed in the brain and functions as a transporter in the biogenesis of HDL by working together with cellular lipid and helical apolipoproteins (Tanaka et al., 2011). Data from several GWAS studies indicate *ABCA7* is a genetic risk factor for LOAD (Hollingworth et al., 2011; Lambert et al., 2013). According to a meta-analysis published on the AlzGene website in April, 2011^2^, a positive association between *ABCA7* rs3764650 and AD was found in total 31011 cases and 48354 controls in all populations. Additionally, several other genetic studies further confirmed the relevance between *ABCA7* SNPs and methylation changes with AD (Yu et al., 2015).

Loss of *ABCA7* in mice is not embryonic lethal, suggesting that ABCA7 is not essential (Kim et al., 2005). However, loss of ABCA7 in mice seems to impair the ability of bone marrow-derived macrophages to uptake oliomeric Aβ. A recent study further showed that crossing between *ABCA7*-deficient and transgenic amyloidogenic mice would double the insoluble Aβ levels and amyloid plaques in the brains of their progenies compared with controls (Li et al., 2015). These findings indicate that ABCA7 may participate in the regulation of Aβ homoeostasis in the brain.

#### CLU

The *CLU* gene is located at 8p21.1 and encodes a multifunctional chaperone protein, clusterin (Wong et al., 1994), which has been implicated in AD for the past 20 years (May et al., 1990; Oda et al., 1994; Calero et al., 2000). Clusterin, also meaning as apolipoprotein J (APOJ), is one of the major apolipoproteins, with upregulated expression in the cortex and hippocampus of AD patients (May et al., 1990; Oda et al., 1994; Pasinetti, 1996). In terms of cholesterol metabolism, clusterin takes part in reverse cholesterol transport as a component of HDL particles (Wollmer, 2010). In addition, clusterin levels have been shown to be elevated in AD plasma (Jones et al., 2010). Meta analysis show that SNPs rs11136000, rs2279590, rs7012010, rs7982, and rs9331888 in *CLU* are protective genetic factors in LOAD^3^. However, the reproducibility of these associations was questionable when ethnic factors were taken into account (Li et al., 2011; Klimkowicz-Mrowiec et al., 2013; Tan L. et al., 2013). Genetic heterogeneity may be the underlying cause at play. Clusterin has several functions similar to apolipoprotein E and there are some interactions between them (Wollmer, 2010). Clusterin can also bind Aβ and modulate Aβ metabolism which are influenced by the molar ratios of clusterin and Aβ. (Yerbury et al., 2007; Aiyaz et al., 2012). In addition, clusterin participates in cell apoptosis and complement regulation, lipid transport and membrane protection, thus plays a role in AD pathogenesis (Bell et al., 2007; Nuutinen et al., 2009; Wollmer, 2010; Martin et al., 2014).

#### SORL1

The *SORL1* gene, also known as *SORLA1* or *LR11*, is situated in 11q23.2–q24.2 and encodes the sortilin-related receptor containing LDL receptor class A repeats (Wollmer, 2010). SORL1 is a member of the VPS10 receptors family which functions by binding lipoproteins including APOE-containing particles, thus mediating endocytotic uptake (Willnow et al., 2008; Wollmer, 2010). The decreased SORL1 expression was found to be associated with AD in 10 years ago (Scherzer et al., 2004). Utilizing microarray screening and immunohistochemistry, researchers showed that AD patients tend to have moderately lower *SORL1* DNA transcription levels in their lymphoblast and significantly decreased SORL1 protein level in their brains, especially the pyramidal neurons and frontal cortex (Scherzer et al., 2004). The suppression of SORL1 expression can lead to overexpression of Aβ and an increased risk of AD (Andersen et al., 2005; Offe et al., 2006; Vardarajan et al., 2012). In addition, two specific clusters of SNPs in *SORL1* were identified to have an association with familial and sporadic AD (Bettens et al., 2008; Lee et al., 2008; Kimura et al., 2009; Tan et al., 2009; Sweet et al., 2010; Reitz, 2013).

Since cholesterol is an integral component of biomembrane, due to the key roles of biomembrane in transportation and cleavage of APP, aggregation of Aβ, and Aβ toxicity, it is entirely possible that abnormality of cholesterol metabolism may have an impact on multiple links of the pathogenic signaling pathways of AD. Epidemiological studies showed that high cholesterol levels in mid-life may lead to dementia in later life. Cholesterol-lowering reagents, such as 3-hydroxy-3-methylglutaryl-coenzym, which is a reductase inhibitor known as statins, may reduce the likelihood of developing dementia. The APOE plays an indispensable role in cholesterol transport of the brain. As a risk factor of AD, the *APOE* gene bridges the gap between AD pathogenesis and cholesterol metabolism. This bridge was further reinforced when recent GWAS studies showed a new batch of genes, including *ABCA7, CLU*, and *SORL1*, may increase the risk of LOAD by affecting cholesterol metabolism.

## Cell Adhesion and Endocytosis

Endocytosis is central to AD because APP, Aβ, and APOE are all internalized through the endolysosomal trafficking pathway, and alterations in APP trafficking through intracellular compartments can directly influence APP proteolytical cleavage (Huang and Mucke, 2012). Several genes identified in GWAS-LOAD studies are associated with cell adhesion and endocytosis, including *BIN1, CD2AP, EPHA1, PICALM*, and *SORL1* (Harold et al., 2009; Hollingworth et al., 2011; Naj et al., 2011; Lambert et al., 2013; Zhang et al., 2015).

### BIN1

The *Bridging Integrator 1* (*BIN1*) is located on chromosome 2q14.3 and has 20 exons which can be spliced into multiple isoforms (Panza et al., 2012; Prokic et al., 2014). BIN1 isoforms, such as isoforms 1–6, are mainly expressed in the brain, in neurons (Prokic et al., 2014). BIN1 was initially found as a tumor suppressor with a MYC-interacting domain, a C-terminal SH3 domain, and an N-terminal BAR (Bin1/Amphiphysin/RVS167) domain (Sakamuro et al., 1996). Processing diverse cellular functions, BIN1 is a key regulator within a cell. From endocytosis to membrane recycling, from cell cycle progression to apoptosis, we can see its roles (Prokic et al., 2014). Cytoskeleton regulation and DNA repair are also involved (Prokic et al., 2014).

*BIN1* was regarded as the second most important genetic risk factor for LOAD after the *APOE 𝜀4*.^4^ Common variants in the *BIN1* gene are initially identified to be associated with AD in GWAS-LOAD studies (Harold et al., 2009; Seshadri et al., 2010; Naj et al., 2011). The main associated SNPs are in the 5′ region, including the most significant SNPs rs744373 and rs7561528, which are located approximately 30 and 25 kb from the *BIN1* coding region, respectively (Harold et al., 2009; Seshadri et al., 2010; Naj et al., 2011; Karch and Goate, 2015). BIN1 can interact with cytoplasmic linker protein 170 (CLIP-170), a microtubule-associated protein (Meunier et al., 2009). Genetic variants in *BIN1* were associated with magnetic resonance imaging measures associated with AD including entorhinal cortex thickness and temporal pole cortex thickness (Biffi et al., 2010). Recent studies have demonstrated the physical interaction between BIN1 and tau protein in human neuroblastoma cells overexpressing these two proteins and in wild type mouse brain homogenates (Kingwell, 2013). Besides its potential effects on tau pathology, BIN1 has also been identified as a regulator of endocytosis and trafficking, immunity and inflammation of the brain, transient calcium potentials, and apoptosis (Tan M.S. et al., 2013).

### CD2AP

*CD2AP* (CD2-associated protein) is located on chromosome 6q12. CD2AP is first discovered as a ligand protein interacting with the T-cell-adhesion protein CD2 (Dustin et al., 1998; Wolf and Stahl, 2003). CD2AP is widely expressed, primarily in epithelial and lymphoid cells (Shih et al., 2001). It consists of three N-terminal SH3 domains followed by a proline rich domain (PRD) and a C-terminal coiled-coil domain (Shih et al., 2001). CD2AP has been shown to be involved in signal transduction, podocyte homeostasis and dynamic actin remodeling (Ma et al., 2010). The protein also takes part in membrane trafficking during endocytosis and cytokinesis (Ma et al., 2010). SNPs rs9296559 and rs9349407 in *CD2AP* are associated with increased LOAD risk (Hollingworth et al., 2011; Naj et al., 2011; Chen et al., 2012). Like *PICALM*, the homologs of *CD2AP* have shown to be able to suppress the Aβ toxicity in yeast and *Caenorhabditis elegans* (Treusch et al., 2011). In addition, RNA interference-mediated disruption of *cindr*, the fly ortholog of *CD2AP*, enhances Tau toxicity in Drosophila (Shulman et al., 2014).

### EPHA1

*EPHA1* (EPH Receptor A1) is a gene situated in chromosome 7q34. The encoded EPH Receptor A1 protein is a member of the ephrin family of tyrosine kinase receptors. Proteins of this family modulate cell adhesion by interacting with ephrin ligands on adjacent cells (Sharfe et al., 2008). Ephrin receptors also plays a role in regulating synapse formation and synaptic plasticity (Lai and Ip, 2009). In addition, these ephrin receptors participate in regulating apoptosis of neural progenitor cells (Kullander and Klein, 2002; Kim et al., 2008). The SNP rs11771145 was identified as a protective genetic factor for LOAD (Hollingworth et al., 2011; Naj et al., 2011; Chen et al., 2012). Albeit some research has been made on the function of ephrin receptors, knowledge on the *EPHA1* gene and its role in AD etiology remains to be lacking.

### PICALM

*PICALM* (phosphatidylinositol binding clathrin assembly protein) is a gene situated in 11q14.2, encoding a clathrin adaptor protein which is produced as two main isoforms with 19–21 exons and 7 different known splice variants. *PICALM* was first cloned as a gene fused with AF10 in acute myeloid leukemia (Dreyling et al., 1996; Ando et al., 2013). Whereas *PICALM* is ubiquitously expressed, its homolog *AP180* is exclusively expressed in neuron (Yao et al., 2005). PICALM is implicated in clathrin mediated endocytosis and intracellular trafficking of the synaptic vesicle protein VAMP2 which is necessary for neurotransmitter release at the presynaptic membrane (Tebar et al., 1999; Schnetz-Boutaud et al., 2012). Two SNPs (rs3851179 and rs541458) 5′ to the *PICALM* gene were identified to be associated with reduced LOAD risk in Caucasians (Harold et al., 2009; Lambert et al., 2009; Lambert et al., 2013). However, the reproducibility of these results was questionable when ethnic factor was taken into account (Li et al., 2011; Klimkowicz-Mrowiec et al., 2013; Tan L. et al., 2013). Genetic heterogeneity may be the underlying reason at play. In addition, AD patients with *PICALM* mutants may manifest different imaging features on MRI (Biffi et al., 2010). Hippocampal volume and entorhinal cortex thickness are the two measures affected most prominently (Biffi et al., 2010). Till now, the role of PICALM in AD etiology has not been known. The *YAP1802*, ortholog of *PICALM*, was found as a modifier of Aβ toxicity in a genome-wide screen in yeast (Treusch et al., 2011; Ando et al., 2013). PICALM was also shown to have a protective role for C. elegans and rat cortical neurons against the toxicity of oligomeric Aβ (Treusch et al., 2011). Another finding was that along with adaptor protein 2 (AP2) and APP-CTF, PICALM would be targeted to the autophagosomes to take part in the clearance of APP-CTF (Tian et al., 2013). In other words, PICALM may have a functional role in the clearance of Aβ via autophagy (Tian et al., 2013). In addition, PICALM displayed a specifically co-localization with neurofibrillary tangles in AD cases, suggesting that PICALM may participate in AD tau pathology (Ando et al., 2013).

Endocytosis is an active transportation mechanism to engulf molecules into a cell via vesicles formed by the cell membrane. It is the basis of various neuronal physiological functions, including synaptic vesicle transport and neurotransmitter release. The transportation and amyloidogenic cleavage of APP are interacting with the endocytosis pathway within cells. Thus, abnormal alterations in endocytosis may contribute to AD pathogenesis. Based on this hypothesis, SNPs in genes related to cell adhesion and endocytosis, such as *BIN1, CD2AP, EPHA1, PICALM*, and *SORL1* are very likely to be involved in AD pathogenesis.

## Immune Response

Neuroinflammation is a hallmark of AD (Heneka et al., 2015). Solid evidence have proven the activation of inflammatory pathways in AD pathogenesis (Heneka et al., 2015; Zhang et al., 2015). Common variants in *ABCA7, CD33, CLU, CR1, EPHA1, HLA-DRB5/DRB1, INPP5D, MEF2C*, and *MS4A*, have been found to be associated with immune responses in recent GWAS studies (Harold et al., 2009; Lambert et al., 2009; Seshadri et al., 2010; Hollingworth et al., 2011; Naj et al., 2011; Lambert et al., 2013). Additionally, rare coding variants in *TREM2* gene related to the immune response were identified to increase risk of AD in LOAD (Guerreiro et al., 2013; Jonsson et al., 2013).

### CD33

*CD33* is located on chromosome 19q13.3 and encodes a transmembrane glycoprotein cluster of differentiation 33 (CD33) (Zhang et al., 2014). CD33, which belongs to the sialic acid-binding immunoglobulin-like lectins (Siglecs) family, bears molecular features of immune cell surface receptors that could trigger immune cell–cell interactions (von Gunten and Bochner, 2008). Studies showed that the expression of CD33 was increased in AD brains (Karch et al., 2012). The rs3865444 in *CD33* was reported to be linked to a lowered LOAD risk (Hollingworth et al., 2011; Naj et al., 2011). The rs3865444 A allele is associated with the decreased overall CD33 expression and an increased proportion of the CD33 isoform lacking exon 2 (Malik et al., 2013). The exon 2 in *CD33* codes the IgV domain which mediates Siglecs family members binding to sialic acid, resulting in inhibition of phagocytosis (Villegas-Llerena et al., 2016). Loss of exon2 of CD33 in microglia abolishes the inhibitory effect of Aβ phagocytosis (Malik et al., 2013). In the context of the rs3865444 risk allele, there are increased cell surface expression of CD33 in monocytes, decreased internalization of Aβ42 accumulation in neuritic and fibrillar amyloid pathology, and more microglias activated (Bradshaw et al., 2013). Thus, CD33 may play an important role in Aβ clearance mediated by microglia in AD brain.

### CR1

*CR1* (Complement receptor 1) is located on chromosome 1q32 and encodes a multifunctional glycoprotein, expressed on microglia and blood cells such as erythrocytes (Villegas-Llerena et al., 2016). CR1 is a cell surface receptor that has binding sites for complement factors C3b and C4b. It participates in the clearance of immune complexes and regulates complement activation (Dunkelberger and Song, 2010). Two SNPs (rs6656401 and rs3818361) in *CR1* have been found to be associated with LOAD risk in most Caucasians (Lambert et al., 2009). These associations could not be reproduced in other ethnic groups including African American, Israeli-Arab, Caribbean Hispanic, and Polish individuals due to the genetic heterogeneity (Li et al., 2011; Klimkowicz-Mrowiec et al., 2013; Tan L. et al., 2013). Genetic variants in *CR1* can affect magnetic resonance imaging measures associated with AD such as entorhinal cortex thickness (Biffi et al., 2010). The exact function of CR1 in AD pathogenesis remains to be elusive. Since Aβ oligomers can bind C3b, some researchers postulated that CR1 may take part in the clearance of Aβ (Crehan et al., 2012).

### HLA-DRB5/DRB1

The *HLA-DRB5/DRB1* locus is a highly polymorphic region located on chromosome 6, encoding a member of the major histocompatibility complex class II (MHC II), which is involved in the immune response and histocompatibility (Trowsdale and Knight, 2013; Villegas-Llerena et al., 2016). Recently, *HLA-DRB5/DRB1* has been shown to be associated with multiple sclerosis and Parkinson’s disease (PD) (International Multiple Sclerosis Genetics Consortium et al., 2011; International Parkinson Disease Genomics Consortium et al., 2011). Although PD and AD have distinct etiologies, they are both characterized by neurodegeneration resulting from abnormal protein aggregation. Therefore, it is a distinct possibility that HLA genes may play a similar role in both PD and AD through regulating inflammatory responses.

### INPP5D

The *INPP5D* gene is a gene situated in chromosome 2q37.1, encoding a 145 kD protein which is a member of the inositol polyphosphate-5-phosphatase (INPP5) family, also known as SH2 domain containing inositol-50-phosphatase 1 (SHIP1) (Arijs et al., 2012; Zhang et al., 2015). INPP5D is expressed predominantly in the hematopoietic cells (Hazen et al., 2009; Arijs et al., 2012; Zhang et al., 2015). On the cell membrane, the protein takes part in various signaling pathways by hydrolyzing the 5′ phosphate from phosphatidylinositol (3,4,5)-trisphosphate and inositol-1,3,4,5-tetrakisphosphate (Scharenberg et al., 1998). Also, INPP5D plays as a negative regulator in B cell proliferation, chemotaxis and activation, as well as IgE- or IgE + Ag-induced inflammatory cytokine release from mast cells (Sly et al., 2003, 2007; Zhang et al., 2015). More studies are needed to understand the mechanism of how SHIP regulates the immune response and inflammation in the brain.

### MEF2C

MEF2C protein is widely expressed and belongs to the MADS box transcription enhancer factor 2 (MEF2) family of transcription factors. The *MEF2C* gene is located on chromosome 5q14.3. It has been reported that MEF2 acts as a central transcriptional component of the innate immune response in the adult fly (Clark et al., 2013). Therefore, it is possible that MEF2C is involved in the inflammatory process in AD brains.

### MS4A

The *MS4A* locus is located on chromosome 11 and contains at least five genes implicated in immune modulation (Villegas-Llerena et al., 2016). The discovery of the MS4A family owes to their homology to CD20, a B-lymphocyte cell surface molecule. Members of the MS4A family, including MS4A6A, are factors affecting AD pathology (Proitsi et al., 2014). Variations in proxies of rs670139 can increase AD risk (Allen et al., 2012).

### TREM2

The *TREM2* gene maps to chromosome 6p21.1, encoding Triggering Receptor Expressed on Myeloid Cells 2 (TREM2). TREM2 is mainly expressed on myeloid cells (Colonna, 2003; Jin et al., 2014). In the brain, TREM2 is primarily expressed on microglia (Lue et al., 2015). TREM2 takes part in inflammatory responses regulation (Rohn, 2013).

Homozygous mutations in *TREM2* gene cause Nasu–Hakola disease, characterized by early onset frontotemporal-like dementia and bone involvement (Klunemann et al., 2005). In addition, some families with FTD-like dementia with leukodystrophy but without bone involvement have homozygous *TREM2* mutations (Guerreiro et al., 2013). Recently, rare variants of the *TREM2* gene have been identified to increase susceptibility to LOAD with an odds ratio similar to that of *APOE 𝜀4* (Boutajangout and Wisniewski, 2013). rs75932628 is the most common variant in *TREM2* polymorphism. It replaces Arginine 47 with Histidine and causes a 3-fold increase in the susceptibility to LOAD (Guerreiro et al., 2013; Jonsson et al., 2013; Zhang et al., 2015). The status of *TREM2* as a major LOAD risk locus was further strengthened by the odds ratio of 3.4 reported in a meta analysis (Guerreiro et al., 2013). The exact functions of TREM2 are not well understood. TREM2 may affect AD pathology through regulating phagocytosis (Hickman and El Khoury, 2014). The expression levels of TREM2 are upregulated in microglia found at the border of amyloid plaque deposits in transgenic AD mice (Lue et al., 2015). Moreover, there was a positive correlation between TREM2 expression and the phagocytic clearance of Aβ in APP transgenic mice (Lue et al., 2015).

Increasing evidence suggests the activation of inflammatory pathways in AD pathogenesis. GWAS suggests that several genes (*ABCA7, CD33, CLU, CR1, EPHA1, HLA-DRB5/DRB1, INPP5D, MEF2C*, and *MS4A*) regulating clearance of misfolded proteins mediated by glia and the inflammatory reaction could increase the risk of AD in LOAD. Furthermore, a rare variant of the *TREM2* gene, with an odds ratio similar to that of *APOE 𝜀4*, was recently identified to be able to increase patients’ susceptibility to LOAD. These results together argue for the point that neuroinflammation is associated with AD pathogenesis. Although there is a lack of understanding how inflammation in AD is affected by these genes, the discovery of them have broadened our knowledge scope of AD and may expedite the unraveling of new therapeutic targets for the prevention and treatment of AD.

## Tau Metabolism

The microtubule-associated protein tau is integral to the pathogenesis of AD. Rare mutations in the *MAPT* gene cause familial dementia syndromes (Lee and Leugers, 2012). GWAS studies have identified several genes that might be potential risk factors for LOAD, including *BIN1, CD2AP, CELF1, FERMT2* and *PICALM*, which are involved in modulating tau neurotoxicity (Harold et al., 2009; Seshadri et al., 2010; Hollingworth et al., 2011; Naj et al., 2011; Lambert et al., 2013).

### CELF1

The *CELF1* gene is located on chromosome 11p11.2 and encodes the CUGBP and Elav-like family member 1 protein (CELF1). Members of the CELF protein family regulate alternative splicing, editing, and translation of mRNA (Wagnon et al., 2012). *CELF1* gene may have a role in myotonic dystrophy type 1 (DM1) because of its interactions with the dystrophia myotonica-protein kinase (DMPK) gene (Roberts et al., 1997). In addition, overexpression of *CELF1* suppressed the neurodegenerative eye phenotype in a transgenic fly model of fragile X-associated tremor/ataxia syndrome (FXTAS) (Sofola et al., 2007). The CELF1 protein modulates rCGG-mediated toxicity via a specific interaction with hnRNP A2/B1 (Sofola et al., 2007). Like *FERMT2*, RNA interference-mediated disruption of *aret*, the fly ortholog of *CELF1*, enhances Tau toxicity in a *Drosophila* model of AD (Shulman et al., 2014).

### FERMT2

The *FERMT2* (Fermitin Family Member 2) gene is located on chromosome 14q22 and is also known as mitogen-inducible gene 2 (*MIG2*) or kindlin 2 (*KIND2*) (Siegel et al., 2003). FERMT2 is ubiquitously expressed in mammalian cells and functions as a kind of cell-extracellular matrix (ECM) structures (Tu et al., 2003). A recent research validated the association of *FERMT2* with AD risk by using a *Drosophila* model (Shulman et al., 2014). RNA interference-mediated disruption of *FERMT2* homologs enhances Tau toxicity in Drosophila indicates these associations (Shulman et al., 2014).

Comprising of hyper-phosphorylated and aggregated tau protein, NFTs are one of the major pathological signatures of the AD brain. The neurotoxicity of Tau plays a central role in AD pathogenesis by affecting Aβ metabolism. It has been shown that there was a causal relationship between certain mutations of either *APP* or *MAPT* and familial dementia syndromes. As more and more genes related to tau neurotoxicity were identified as risk genes of AD, hopefully the molecular basis between Tau toxicity and AD would gradually become clear.

## Perspectives

Genome-wide association studies is a powerful tool in identifying putative genetic risk factors. To date, more than 20 genetic variants have been identified as risk factors of AD. There is no gainsaying that GWAS helps us find novel perspectives on the pathogenesis of AD. However, there are still some limitations to be scrutinized. Firstly, some of these AD-associated variants are too rare or too weak to be used as prognostic predictors, which to some extent confound the integration of potential pathophysiological pathways of AD. On the contrary, whole exome sequencing has also discovered rare variants, such as *TREM2* variants, whose odds ratios are comparable to that of *APOE 𝜀4* in terms of increasing the risk of AD. Therefore, the range of these variants seems to be overly wide, which may have made it difficult for us to form a coherent and integrated theory. Moreover, although both SNPs with minor allele frequency down to 1% and novel functional exonic variants have been incorporated into the latest version of GWAS arrays, the detection would still be problematic when it comes to variants not tagged by the known SNPs or some extremely rare structural variants whose minor allele frequency are less than 1%. However, the role of such rare and structural variants should not be negligible in complex disease like AD. Therefore, ongoing and future large-scale next-generation whole exome or whole genome sequencing techniques need to address the issues aforementioned to accurately target causative variants in regions identified by GWAS. For only truly causative variants could yield meaningful functional studies to dissect molecular pathways in AD pathogenesis (**Table 1**).

**Table 1:** Potential mechanisms of AD genes.

Gene	SNP	Chromosome position	Protein	EOAD/ LOAD	Proposed function	Implicated pathways
*ABCA7*	rs3764650 rs4147929	19p13.3	ATP-binding cassette transporter A7	LOAD	Lipid homeostasis	Cholesterol metabolism; immune response
*ADAM10*	rs61751103 rs145518263	15q21.3	A disintegrin and metalloprotease family, AMAD10	LOAD	Proteolytic cleavage of integral membrane proteins	Aβ metabolism
*APOE*	rs429358 rs7412	19q13.2	Apolipoprotein E	LOAD	Mediates binding, internalization, and catabolism of lipoproteins	Cholesterol metabolism
*APP*	–	21q21.3	Amyloid precursor protein	EOAD	Neurite outgrowth, adhesion, and axonogenesis	Aβ metabolism
*BIN1*	rs744373 rs7561528	2q14	Bridging Integrator 1	LOAD	Regulation of endocytosis of synaptic vesicles	Cell adhesion and endocytosis; tau metabolism
*CASS4*	rs7274581	20q13.31	Cas scaffolding protein family member 4	LOAD	Docking protein in tyrosine-kinase signaling involved in cell adhesion and spreading	Cytoskeleton and axonal transport
*CD2AP*	rs9296559 rs9349407	6p12	CD2-associated protein	LOAD	Scaffold molecule regulating actin cytoskeleton	Cell adhesion and endocytosis; tau metabolism
*CD33*	rs3865444	19q13.3	Cluster of differentiation 33	LOAD	Mediates sialic acid-dependent binding to cells	Immune response
*CELF1*	rs10838725	11p11	CUGBP and Elav-like family member 1	LOAD	Regulates pre-mRNA splicing	Tau metabolism
*CLU*	rs11136000, rs2279590, rs7012010, rs7982, rs9331888	8p21-p12	Clusterin	LOAD	Chaperone; regulation of cell proliferation	Cholesterol metabolism Immune response
*CR1*	rs6656401 rs3818361	1q32	Complement receptor 1	LOAD	Mediates cellular binding of immune complexes that activate complement	Immune response
*DSG2*	rs8093731	18q12.1	Desmoglein 2	LOAD	Mediates cell–cell junctions between epithelial and other cell type	Cytoskeleton and axonal transport
*EPHA1*	rs11771145	7q34	EPH Receptor A1	LOAD	Brain and neural development; angiogenesis, cell proliferation, and apoptosis	Cell adhesion and endocytosis; immune response
*FERMT2*	rs17125944	14q22.1	Fermitin Family Member 2	LOAD	Actin assembly and cell shape and mediator of angiogenesis	Tau metabolism
*HLA-DRB5/DRB1*	rs9271192	6p21.3	Major histocompatibility complex, class II, DR beta 5- DR beta 1	LOAD	Immunocompetence and histocompatibility	Immune response
*INPP5D*	rs35349669	2q37.1	Inositol polyphosphate-5-phosphatase	LOAD	Negative regulator of myeloid cell proliferation and survival	Immune response
*MEF2C*	rs190982	5q14.3	Myocyte enhancer factor 2C	LOAD	Controls synapse formation	Immune response
*MS4A4*/*MS4A6E*	rs983392 rs670139	11q12.1	Membrane-spanning 4-domains, subfamily A, member 4A/6E	LOAD	Signal transduction	Immune response
*NME8*	rs2718058	7p14.1	NME/NM23 family member 8	LOAD	Ciliary functions	Cytoskeleton and axonal transport
*PICALM*	rs3851179 rs541458	11q14	Phosphatidylinositol binding clathrin assembly protein	LOAD	AP2-dependent clathrin-mediated endocytosis	Cell adhesion and endocytosis; tau metabolism
*PSEN1*	–	14q24.3	Presenilin 1	EOAD	Component of catalytic subunit of gamma-secretase complex	Aβ metabolism
*PSEN2*	–	1q31-q42	Presenilin 2	EOAD	Component of catalytic subunit of gamma-secretase complex	Aβ metabolism
*PTK2B*	rs28834970	8p21.1	Protein tyrosine kinase 2 beta	LOAD	Induction of long term potentiation in hippocampus	Endocytosis
*SLC24A4/RIN3*	rs10498633	14q32.12	Solute carrier family 24, member 4/ Ras and Rab interactor 3	LOAD	Brain and neural development	Neural development, synapse function, endocytosis
*SORL1*	rs11218343	11q23.2-q24.2	Sortilin-related receptor containing LDL receptor class A repeats	LOAD	APOE receptor; binds LDL and RAP and mediates endocytosis of the lipids to which it binds	Cholesterol Metabolism; Cell adhesion and endocytosis
*TREM2*	rs75932628	6p21.1	Triggering Receptor Expressed on Myeloid Cells 2	LOAD	Induces phagocytosis of apoptotic neurons, and regulates Toll-like receptor mediated inflammatory responses, and microglial activation	Immune response
*ZCWPW1*	rs1476679	7q22.1	Zinc finger, CW type with PWWP domain 1	LOAD	Epigenetic regulation	Epigenetic regulation

## Conclusion

AD is a complex disorder. What we have known is still a drop in the ocean. To improve the prevention and treatment strategies of AD, finding the potential genes in AD pathogenesis and their relationships is a necessary and essential step. It is the fundamental basis for the molecular diagnosis of AD and the mechanistic study on neurodegeneration. Current genetic findings indicated putative disease mechanisms including Aβ metabolism, cell adhesion and endocytosis, immune response, tau metabolism. Future GWASs or next generation sequencing (NGS) approaches studies would keep playing important roles in revealing promising therapeutic targets.

## Author Contributions

QS, RL, and YS discussed the concepts and wrote the manuscript. QS, NX, and BT revised the manuscript.

## Conflict of Interest Statement

The authors declare that the research was conducted in the absence of any commercial or financial relationships that could be construed as a potential conflict of interest.

## Footnotes

1 http://www.molgen.ua.ac.be/ADmutations

2 www.alzgene.org

3 http://www.alzgene.org

4 http://www.alzgene.org/
